# The Zambian Preterm Birth Prevention Study (ZAPPS): Cohort characteristics at enrollment

**DOI:** 10.12688/gatesopenres.12820.3

**Published:** 2019-07-15

**Authors:** Marcela C. Castillo, Nurain M. Fuseini, Katelyn Rittenhouse, Joan T. Price, Bethany L. Freeman, Humphrey Mwape, Jennifer Winston, Ntazana Sindano, Courtney Baruch-Gravett, Benjamin H. Chi, Margaret P. Kasaro, James A. Litch, Jeffrey S.A. Stringer, Bellington Vwalika

**Affiliations:** 1 University of North Carolina at Chapel Hill, Chapel Hill, NC, USA; 2 UNC Global Projects Zambia, Lusaka, Zambia; 3 Global Alliance for the Prevention of Prematurity and Stillbirth, Seattle, WA, USA; 4 University of Zambia School of Medicine, Lusaka, Zambia

**Keywords:** Preterm Birth, Africa, Cohort

## Abstract

**Background: **Sub-Saharan Africa bears a disproportionate burden of preterm birth and other adverse outcomes. A better understanding of the demographic, clinical, and biologic underpinnings of these adverse outcomes is urgently needed to plan interventions and inform new discovery.

**Methods: **The Zambian Preterm Birth Prevention Study (ZAPPS) is a prospective observational cohort established at the Women and Newborn Hospital (WNH) in Lusaka, Zambia. We recruit pregnant women from district health centers and the WNH and offer ultrasound examination to determine eligibility. Participants receive routine obstetrical care, lab testing, midtrimester cervical length measurement, and serial fetal growth monitoring. At delivery, we assess gestational age, birthweight, vital status, and sex and assign a delivery phenotype. We collect blood, urine, and vaginal swab specimens at scheduled visits and store them in an on-site biorepository. In September 2017, enrollment of the ZAPPS Phase 1—the subject of this report—was completed. Phase 2, which is limited to HIV-uninfected women, reopened in January 2018.

**Results: **Between August 2015 and September 2017, we screened 1784 women, of whom 1450 (81.2%) met inclusion criteria and were enrolled. The median age at enrollment was 27 years (IQR 23–32) and median gestational age was 16 weeks (IQR 13–18). Among women with a previous pregnancy (n=1042), 19% (n=194) reported a prior miscarriage.  Among parous women (n=992), 41% (n=411) reported a prior preterm birth and 14% (n=126) reported a prior stillbirth. The HIV seroprevalence was 24%.

**Discussion: **We have established a large cohort of pregnant women and newborns at the WNH to characterize the determinants of adverse birth outcomes in Lusaka, Zambia. Our overarching goal is to elucidate biological mechanisms in an effort to identify new strategies for early detection and prevention of adverse outcomes. We hope that findings from this cohort will help guide future studies, clinical care, and policy.

## Introduction

Preterm birth is a global challenge impacting both developed and developing countries ^1, 2^. It contributes to approximately 35% of neonatal and 75% of perinatal mortality each year ^3, 4^. Further, preterm infants who survive are at an elevated risk of long-term respiratory, cardiovascular, gastrointestinal, and neurodevelopmental morbidities. These complications may affect subsequent health, growth, psychosocial functioning, and even economic capacity of these individuals ^5– 7^.

The greatest burden of mortality and morbidity from preterm births occurs in low- and middle-income countries (LMICs) ^8^. Of an estimated 14.9 million preterm births globally each year, 13.6 million (91%) occur in LMICs ^1^. Preterm birth rates are as low as 5% in some European countries and as high as 18% in some African countries ^1^, precisely where the resources to prevent preterm birth and manage preterm infants are least developed. In Zambia, for example, the preterm birth rate is estimated to be 13% ^1^. Each year there are 77,600 preterm births and 6,800 infant deaths due to preterm birth complications ^9^.

The burden of maternal HIV infection is also high in many LMIC settings, where it has been associated with a 50% increased risk of preterm birth ^10^. Although the increasing availability of maternal antiretroviral therapy has led to dramatic reductions in pediatric HIV incidence ^11^, it does not seem to reduce HIV-attributable preterm birth in this population. In fact, antiretroviral drug exposure may in fact increase the risk of preterm birth among some HIV-infected gravidas ^12– 14^.

Preterm birth can result from many different etiological entities. Approximately one-third of preterm deliveries are indicated because of pre-eclampsia, hemorrhage, abnormal placentation, intra-uterine growth restriction, oligohydramnios, or multi-fetal gestation. Spontaneous preterm labor is implicated in about 40% of preterm births; another 25% are related to preterm prelabor rupture of the membranes ^15– 17^. The underlying causes of spontaneous preterm birth in HIV-infected and HIV-uninfected populations are not well understood. Although several maternal and newborn interventions (e.g. antenatal corticosteroids, neonatal resuscitation, and kangaroo mother care) can reduce the complications of preterm birth, prevention is key. Much remains to be discovered about the risk factors, causes, and pathophysiology of preterm delivery, and how to prevent its occurrence.

## Methods

### Study design

The Zambian Preterm Birth Prevention Study (ZAPPS) aims to establish a well-characterized pregnancy cohort to better understand the risk factors associated with preterm birth and other adverse birth outcomes in a LMIC setting. The cohort was established to be a local resource to the University of Zambia School of Medicine and to contribute to general scientific knowledge around the biology of pregnancy and parturition.

ZAPPS enrolls pregnant women at the Women and Newborn Hospital of the University Teaching Hospital (UTH) in Lusaka, Zambia into a prospective antenatal cohort. The UTH is the province’s only tertiary referral center, serving a primary catchment population of approximately 2 million people. The Women and Newborn Hospital receives referrals for high-risk pregnancies, including those with complex medical histories, history of prior preterm birth, stillbirth, or pregnancy loss, and has a very busy labor ward with approximately 18,000 deliveries per year ^18^. Study participants are recruited from the UTH and five nearby high-volume Lusaka district health clinics. We established this cohort with the aim to better characterize demographic determinants, biomedical causes, and underlying pathophysiologic mechanisms associated with adverse birth outcomes in Lusaka, Zambia. Through the Global Alliance to Prevent Prematurity and Stillbirth, we collaborate with a consortium of international scientists, many of whom are also working in LMIC countries, in order to advance our understanding of the causes of preterm delivery. This study was designed in accordance with the Strengthening The Reporting of Observational Studies in Epidemiology (STROBE) guidelines ^19^.

### Study participants

Pregnant women who meet the following criteria are eligible for enrollment in ZAPPS: (1) 18 years of age or older; (2) viable intrauterine singleton or twin pregnancy; (3) presentation to antenatal care prior to 20 weeks’ gestation if HIV-uninfected or 24 weeks’ if HIV-infected; (4) residing within Lusaka with no plans to relocate during the study follow-up period; (4) willing to provide written, informed consent; (5) willing to allow participation of their infant(s) in the study; (6) willing to be followed up at home for birth outcomes if necessary. Initially, all pregnant women with a gestational age ≥ 20 weeks by a standard algorithm ^20^ were excluded; however, this criterion was extended to ≥24 weeks for HIV-infected women following a protocol amendment in July 2016 to align this group with related, ongoing clinical trials at this site ( NCT03297216, NCT02970552).

In September 2017, enrollment of the ZAPPS Phase 1 – the subject of this report – was completed and enrollment paused as we amended the protocol to focus on HIV-uninfected women only. Phase 2 reopened enrollment in January 2018.

### Primary objective

The primary objective of this study is to establish a well-characterized cohort of pregnant women and their infants – and an accompanying specimen biorepository – with follow-up through delivery and up to 42 days postpartum. Our overarching goal is to create a resource in Zambia to better elucidate the biological mechanisms leading to preterm delivery in an effort to identify new strategies for early detection and prevention.

### Outcomes

The primary outcome of the ZAPPS cohort study is preterm birth, defined as birth prior to 37 weeks completed gestation. The secondary outcomes are: (1) delivery prior to 34 weeks completed gestation and (2) low birth weight, defined as infants weighing less than 2500 grams at delivery.

### Study procedures

Potential participants are identified at an early antenatal visit. Community educators approach participants who may be preliminarily eligible by gestational age criteria based on reported last menstrual period and fundal height. Potentially eligible women are escorted to the UTH study clinic. After an information session, a sonographer performs an ultrasound examination to determine fetal viability and gestational age estimation either by crown rump length (if <14 weeks) ^21^ or fetal biometry (if ≥14 weeks) measurements ^22^. Women deemed eligible for study participation and choosing to participate are administered an informed consent in the language of their choice: English, Nyanja, or Bemba. While screening and enrollment procedures may occur on the same day, women could return on a subsequent day for enrollment to allow time to consider the risks and benefits of study participation and to discuss the study with their family.

### Clinical care and follow-up

Study participants receive routine antenatal care at the ZAPPS study clinic at the UTH, with visits scheduled at enrollment, 24 weeks, 32 weeks, and 36 weeks of gestation, according to standard of care in Zambia. Additionally, women are asked to return to the clinic for a postpartum visit, typically 6 weeks after delivery ( Table 1).

**Table 1.** Schedule of events, demographic and clinical data collected among all participants in ZAPPS cohort.

Gestational age (weeks)	<20 ^	20-22	24	28 ^†^	32	34 ^†^	36	Delivery	42 days
ADMINISTRATIVE/REGULATORY PROCEDURES									
Informed consent	•								
Collection/review of locator info	•	•	•	•	•	•	•	•	•
COLLECTION OF DEMOGRAPHIC VARIABLES									
Age, education, socioeconomic status	•								
Substance use	•		•		•		•		
Marital status	•								
Pregnancy intention	•								
Sexual health	•								
Vaginal practices	•								
Intimate partner violence screening	•								
Nutritional assessment	•								
Maternal depression screen			•						•
OBSTETRICAL ULTRASOUND PROCEDURES									
Dating biometry ultrasound	•								
Fetal biometry ultrasound					•				
Cervical length ultrasound		•		•		•			
MATERNAL CLINICAL HISTORY AND PHYSICAL EXAM									
Obstetrical history	•								
Medical history	•		•		•		•	•	•
Maternal height & weight	•		•		•		•	•	•
Maternal mid-upper arm circumference	•		•		•		•	•	•
Maternal vital signs	•		•		•		•	•	•
Maternal physical exam	•		•		•		•	•	•
Fetal heart rate	•		•		•		•	•	
Fundal height	•		•		•		•		
Fetal lie	•		•		•		•	•	
INFANT CLINICAL HISTORY AND PHYSICAL EXAM									
Neonatal physical exam								•	
Neonatal vital status / APGAR								•	
Newborn assessment								•	
Infant physical exam									•
Infant feeding status assessment									•
Infant HIV diagnostic assessment (if exposed)								•	•
LABORATORY PROCEDURES									
Maternal HIV (rapid EIA)	•				•				
Maternal syphilis (RPR)	•				•				
Maternal malaria	•								
Maternal hemoglobin (hemocue)	•				•				
Maternal urinalysis (& culture if +)	•	•	•	•	•	•	•		
SPECIMEN COLLECTION FOR STORAGE / FUTURE TESTING									
Vaginal ± rectal swab storage	•		•		•			•	
Blood storage	•		•		•			•	
Urine storage	•		•		•			•	
Placenta, membranes, cord histopathology, and storage								•	
Infant blood sample via heel prick								•	

^^^ Extended to <24 weeks for HIV-infected in July 2016 ^†^ Additional visits for participants with short cervix * Birth weight, birth length, head circumference, foot length, physical and neuromuscular maturity

After enrollment, all participants return for universal cervical length evaluation between 20 and 24 weeks’ gestation. Those with a short cervix by transvaginal ultrasound, defined as <2.5 cm ^23^, then attend additional visits scheduled at 28 and 32 weeks’ gestation for repeat cervical length ultrasounds and are referred to a study physician at the UTH for further counseling and follow-up. All participants undergo an additional fetal biometry ultrasound, performed at 32 weeks’ gestation. Each study sonographer is trained using curricula adapted from the INTERGROWTH-21 ^21, 24^ and Cervical Length Education and Review ( CLEaR) program for cervical length measurements. All biometry parameters are measured twice and then averaged. Cervical length – measured three times over a period of 3 to 5 minutes – is first measured by transabdominal ultrasound; those whose transabdominal cervical length is <3.5cm or not measurable then undergo transvaginal measurement ^25, 26^.

At each antenatal care visit, study nurses perform a vital sign assessment and a physical exam, which includes maternal height and weight; mid-upper arm circumference measurement; fundal height measurement; assessment for pallor, edema, and abdominal tenderness; fetal heart rate assessment; and cervical exam as clinically indicated. We use point-of-care tests for HIV, anemia, malaria, syphilis, and urinary tract infection, and provide tetanus toxoid injection(s), iron, folate, malaria intermittent preventive treatment, and de-worming treatment in accordance with local standards of care. Participants with pre-existing or new HIV diagnoses are counseled and referred to appropriate antiretroviral therapy and prevention of mother-to-child transmission services. At the postpartum visit, study nurses assess maternal and infant interval complications, perform maternal and infant physical exams, assess infant feeding and general well-being, and provide health education counselling. The Edinburgh Postnatal Depression Screen is self-administered by participants at 24 weeks and again at the postpartum visit. Participants who screen positive are referred for further care at the UTH outpatient psychiatric clinic.

Throughout the study, study nurses assess participants’ past medical and obstetrical history and current pregnancy signs and symptoms. Participants are asked specifically if they have ever been diagnosed with high blood pressure, heart disease, diabetes, HIV/AIDS, tuberculosis, or any other chronic illness. Study staff carefully screen participants at each study visit for the presence of adverse or serious adverse events. Participants are referred to the appropriate higher level care provider at the UTH for any adverse events identified that require medical care beyond the scope of the study nurses’ practice.

To maximize retention, locator information on all participants is collected at screening and reviewed at each subsequent encounter. All participants are informed during the consent process that their locator sources will be used to contact them if they do not attend their scheduled study visits. Missed visits are identified by an electronic database that tracks expected and actual visits. If a participant misses a scheduled visit, study staff follow standardized procedures to attempt to contact the participant through the following mechanisms: (1) phone contact with the participant directly, (2) phone contact with other contacts provided on the participant’s locator information, and (3) home visits.

### Data collection and management

Clinical data: After enrollment, study staff collect medical, antenatal, and HIV history data (as applicable) through interviewer-administered questionnaires and review of participants’ medical records. At the time of delivery, or at first contact postpartum, detailed information is collected about the clinical course of the participant’s delivery and delivery outcomes for both the mother and infant(s). This allows clinical phenotyping of all adverse birth outcomes. Shortly after delivery and prior to hospital discharge, the study team documents assessment of infant vital signs, weight, length, head circumference, complete physical exam, and of neuromuscular maturity using the New Ballard Score ^27^. If the infant requires admission to the neonatal intensive care unit, the newborn assessment is done after the infant is deemed stable by the pediatrician.

Biological specimen collection: Trained study nurses collect maternal specimens at enrollment, 24-week, and 32-week visits, as well as at delivery ( Table 1) following approved standard operating procedures to ensure quality and uniformity. While the 24- and 32-week study visits are scheduled according to gestational age, in the event that a participant misses her appointment, specimens may be collected as soon as possible once she returns to clinic. Trained study nurses collect maternal specimens pre-delivery as well as placenta and cord blood specimens immediately following delivery. All specimens are stored and transferred in insulated containers with continuous temperature monitoring to the on-site lab by clinic staff within two hours of collection. Study lab staff process all specimens according to assay manufacturers’ instructions, analyzing some specimens immediately per standard antenatal care guidelines and storing others for later study-related analyses ( Table 1). HIV-1 plasma viral loads are performed for participants identified as HIV-infected at enrollment. Lab staff follow strict quality protocols for maternal blood processing to produce aliquots of whole blood, serum, plasma, and buffy coat for storage in barcoded cryotubes. All specimens are stored at -80°C in temperature-controlled freezer systems equipped with continuous temperature monitoring and text message notification of temperature deviations. The UTH serves as the primary biorepository for stored specimens, with redundancy at a central project repository in Seattle, Washington and at the University of North Carolina at Chapel Hill in Chapel Hill, NC.

### Ethical considerations

The ZAPPS protocol was developed in consultation with a local community advisory board to ensure study procedures are acceptable in the communities from which participants would be recruited. The study and its protocol revisions undergo continuing ethical review by the relevant research ethics authorities at the University of Zambia School of Medicine (Reference number: 016-04-14) and the University of North Carolina School of Medicine (Study number: 14-2113). Participation in all study activities is voluntary, and each participant provides written, informed consent prior to enrollment.

To address the minimal risks associated with participation in this non-interventional study, all study personnel have been trained on standard operating procedures to protect participant privacy and confidentiality. Staff receive protection of human research participants training prior to conducting any study activities and every two years thereafter. Key research staff members complete Good Clinical Practice or Good Clinical Laboratory Practice training, as applicable. All study-related and unrelated adverse events and social harms are graded using the National Institute of Health’s Division of AIDS Table for Grading the Severity of Adult and Pediatric Adverse Events. All adverse events are reported to regulatory authorities, according to their individual guidelines. 

All participants in this study may benefit from enhanced health education and close clinical monitoring. The knowledge generated from this observational study regarding maternal risk factors and neonatal outcomes of preterm birth are expected to outweigh the risks of participation. Conclusions drawn from this knowledge may inform future clinical trials on the prevention of adverse birth outcomes in low-resource settings, which may in turn enable policymakers worldwide to make informed decisions regarding effective interventions for the improvement of maternal-neonatal health.

## Results

### Characteristics of cohort at enrollment

Between August 2015 and September 2017, 1784 women were recruited and screened from local antenatal clinics by ZAPPS study staff. Among them, 1450 (81.3%) met inclusion criteria and were enrolled. Of the 334 not enrolled, 260 (78%) were at advanced gestational age on ultrasound, 18 (5%) had a non-viable pregnancy, 15 (4%) were unwilling to provide informed consent, 10 (3%) were less than 18 years old, 7 (2%) were unwilling to remain in the study area, 3 (1%) were not willing to be contacted by study staff, 2 (1%) were not willing to attend study visits or deliver at UTH, and 19 (6%) were not enrolled for other reasons.

The median age of participants in the cohort is 27 years (IQR 23–32) ( Table 2). Most women (n=1202 of 1437; 84%) are married or cohabiting with their partner, and have been pregnant at least once in the past (n=1042 of 1450, 72%). Among participants with a prior pregnancy, 19% (n=194) reported a prior miscarriage. Among parous participants (n=992), 41% (n=411) reported a prior preterm birth and 14% (n=126) reported a prior stillbirth. Participants are enrolled at a median gestational age of 16 weeks (IQR 13–18); 427 of 1450 (29%) enrolled prior to 14 weeks’ gestational age.

**Table 2.** Baseline characteristics of ZAPPS cohort, N=1450.

Characteristic	N	Value * % or Median (IQR)
Age, years	1409	27 (23,32)
<20	111	7.9
20–34	1116	79.2
≥35	182	12.9
Missing	41	-
Marital status		
Not married and not cohabiting	235	16.4
Married or cohabiting	1202	83.7
Missing	13	-
Education		
None	26	1.8
0–12 years	1225	85.4
≥12 years	184	12.8
Missing	15	
Source of drinking water		
Piped	1340	93.3
Other	96	6.7
Missing	14	-
Toilet facilities in household		
Flush or Pour	762	53.0
Pit or Latrine	673	46.8
Other	2	0.1
Missing	143	-
Floor material in home		
Natural/rudimentary	138	9.6
Finished	1299	90.4
Missing	13	-
BMI, kg/m ^2^	1366	23.6 (21.2,27.2)
<18.5	71	5.2
18.5–30.0	1103	80.8
>30.0	192	14.1
Missing	84	-
GA at enrollment, weeks	1450	16 (13,18)
<14	427	29.4
≥14	1023	70.6
Gravidity	1450	2 (1,4)
Primigravid	408	28.1
Multigravid	1042	71.9
Parity	1450	1 (0,2)
Nulliparous	458	31.6
Parous	992	68.4
Prior miscarriage, n=1042		
Multigravida, no prior miscarriage	848	81.4
Multigravida, ≥1 prior miscarriage	194	18.6
Prior PTB, n=992		
Parous, no prior PTB	581	58.6
Parous, ≥1 prior PTB	411	41.4
Prior SB, n=992		
Parous, no prior SB	780	86.1
Parous, ≥1 prior SB	126	13.9
Missing	86	-
Blood pressure at enrollment		
Normotensive	1367	96.3
Hypertensive ^	52	3.7
Missing	31	-
HIV serostatus at enrollment		
Negative	1097	75.8
Positive	350	24.2
Missing	3	-
Syphilis at enrollment		
Non-reactive	1272	94.8
Reactive	70	5.2
Missing	108	-
Hemoglobin at enrollment, mg/dL	1025	12 (11,13)
≥10.5	885	86.3
<10.5	140	13.7
Missing	425	-
Malaria at enrollment		
Negative	1143	99.6
Positive	5	0.4
Missing	302	-
Urinalysis at enrollment		
Normal	1302	95.0
Abnormal †	69	5.0
Missing	79	-

IQR, interquartile range; BMI, body mass index; GA, gestational age; PTB, preterm birth; SB, stillbirth * Not all columns sum to 100% due to rounding ^ Defined as systolic blood pressure ≥ 140 and/or diastolic blood pressure ≥ 90 † Defined as 1+ leukocyte esterase and/or + nitrites

The baseline HIV seroprevalence in our cohort is 24% (n=350 of 1447), of whom 55% (n=186 of 340) had undetectable viral load. Nearly 4% (n=52 of 1419) of participants had elevated blood pressure (≥140/90) at enrollment; 5% (n=69 of 1371) had a urinalysis consistent with bacteriuria or urinary tract infection (1+ leukocyte esterase and/or nitrites). Syphilis was prevalent in 5% (n=70 of 1342) of our cohort at baseline. Malaria is uncommon in our cohort: 5 of 1148 participants (0.4%) tested positive for malaria by rapid test. 14% (n=140 of 1025) were anemic (Hgb <10.5mg/dL) at baseline.

## Discussion

The underlying pathological processes responsible for activation of the common parturition pathway in preterm labor, preterm prelabor rupture of membranes, and preterm delivery are incompletely understood. A comprehensive investigation of these processes could help define clinical signs and biological markers of high-risk pregnancies and allow the development and application of early preventive interventions targeted to those women at highest risk. Our cohort study will describe the frequency, predictors, and potential confounders of preterm birth and other adverse outcomes in HIV-infected and uninfected women. Our biorepository is being established in parallel to investigate biological correlates of these outcomes.

Future planned analyses of the ZAPPS cohort data will investigate frequency and determinants of the following adverse birth outcomes, both individually and in composite: preterm birth (delivery <37 weeks), very preterm birth (delivery <34 weeks), stillbirth, low birthweight (<2500g), very low birthweight (<1500g), small for gestational age (<10%ile), and very small for gestational age (<3%ile). We will define birthweight-for-age according to INTERGROWTH-21 ^st^ standards ^24^. We will distinguish spontaneous preterm deliveries from provider-indicated preterm deliveries and investigate the prevalence and distribution of preterm phenotypes ^28^, both within our cohort and relative to other studies ^29^. Specimens stored at our UTH laboratory will be used for study-related analyses to identify inflammatory markers (e.g., chemokines, cytokines), microbiome community states, metabolic analytes, proteins, hormones, transcripts, and various infectious factors that may be predict adverse birth outcomes. As nearly one-fourth of our cohort was HIV-infected at enrollment, we will conduct specific analyses within this population to better understand the effect of both HIV and antiretroviral therapy on adverse birth outcomes.

Gestational age can be estimated by patient report of the last normal menstrual period (LMP), ultrasound biometry ^24, 30^, or a combination of the two ^20^. Our experience in Zambia is that the LMP is an imprecise measure that artificially inflates the actual rate of preterm birth in the population ^31^, and that it may in fact introduce bias ^32^. We chose to establish gestational age by ultrasound alone in this cohort.

The strength of ZAPPS is found in its size (nearly 1500 participants enrolled to date) and its design (a prospective antenatal cohort enrolling in early pregnancy). We note that our cohort is at risk of attrition, a well-known challenge of antenatal cohorts, as well as biases of selective participation contributing to a cohort not fully representative of the general population. We enroll pregnant women in the nation’s capital of Lusaka, and we have specifically prioritized the enrollment of HIV-infected women, relaxing the eligibility criteria for gestational age at enrollment for this population. This over-representation of HIV-infected participants in our cohort will enhance our ability to investigate epidemiologic and mechanistic associations of HIV and preterm birth. We acknowledge substantial missingness of key point-of-care results (hemoglobin, syphilis, and malaria) as another important limitation to our cohort. Since our study is integrated with routine care at clinical sites, several participants—particularly in the early conduct of the study—did not undergo repeat testing at the study clinic if they had recently done so at the clinic from which they were recruited. This has been rectified in Phase 2 of the cohort.

In summary, we have established a well-characterized antenatal cohort in Lusaka, Zambia that benefits from ultrasound gestational age dating, longitudinal clinical assessments, biological specimen collection and storage, and careful classification of birth and neonatal outcomes (including phenotyping of all preterm births and stillbirths). The knowledge gained from this study has the potential to drive future research in preterm birth and other adverse birth outcomes, to inform the development of novel preventive therapies and treatments, and to influence clinical care and health policy worldwide.

### Collaboration

The ZAPPS study is part of the Preventing Preterm Birth Initiative of the Global Alliance to Prevent Prematurity and Stillbirth ( GAPPS). The Zambia cohort is co-led by the University of Zambia and the University of North Carolina at Chapel Hill. Study findings will be made available through appropriate local channels, including academic and public health research symposia. Our primary purpose is as a shared resource and we invite collaborators with high-impact ideas to apply for access to data and stored specimens from the ZAPPS study as well as other sites in the GAPPS biorepository network. Potential collaborators are invited to contact GAPPS directly ( info@gapps.org) or the ZAPPS principal investigators: Jeffrey Stringer ( jeffrey_stringer@med.unc.edu) and Bellington Vwalika ( bvwalika@unza.zm).

## Data availability

De-identified individual patient data underlying Table 2 are available on Open Science Framework: http://doi.org/10.17605/OSF.IO/UNE9Y ^33^

Data are available under the terms of the Creative Commons Zero "No rights reserved" data waiver (CC0 1.0 Public domain dedication).
