# Supplementary material for: The Zambian Preterm Birth Prevention Study (ZAPPS): Cohort characteristics at enrollment
Source: Gates Open Res. 2019 Jul 15;2:25. Originally published 2018 May 2. [Version 3] doi: 10.12688/gatesopenres.12820.3 (PMC6350406; doi:10.12688/gatesopenres.12820.3)
Supplement: Peer review history

# Peer review: Reviewer response for version 2

**DOI:** 10.21956/gatesopenres.13986.r26804

**Published:** 2019-01-03

**Authors:** Fyezah Jehan

**Affiliations:** 1 Department of Pediatrics and Child Health, Aga Khan University, Karachi, Pakistan

Thank you for uploading a revised version. The paper has improved greatly in terms of clarity of design and study methods. I approve with no reservations.

I confirm that I have read this submission and believe that I have an appropriate level of expertise to confirm that it is of an acceptable scientific standard.

# Peer review: Reviewer response for version 1

**DOI:** 10.21956/gatesopenres.13888.r26413

**Published:** 2018-05-24

**Authors:** Fyezah Jehan

**Affiliations:** 1 Department of Pediatrics and Child Health, Aga Khan University, Karachi, Pakistan

Overall an important report of a biorepository cohort in LMIC, in a high prematurity setting defining baseline characteristics of the cohort at enrollment. Some recommendations for improvement :-

 Abstract: Needs to be realigned with the article text. Methods incomplete, all salient points need to be mentioned eg. GA at eligibility, samples being stored in biorepository. Similarly, what outcome information is being collected. Conclusion mentions adverse birth outcomes although focus in the introduction has been on preterm.

 Article:

 Introduction: Focus of discussion has been on prematurity its risk factors and causes, while the title, methods, objectives are related to establishment of a bio repository. It isn't entirely clear if this is a description of the biro repository or a description of cohort characteristics of the women in the biorepository or both. A clearer enunciation of the objective needs to be made. Adverse outcomes to be studied are detailed in the discussion session so I would suggest to either bring that detail into the methods, in order to describe the biorepository completely.

 Methods:

 Clinical care and followup/ Data collection and management: Overall lacks specificity in discussion for e.g. what particular medical history and exam is done? How is size, neuromuscular maturity assessed? Is there a window of collection? Who does it? What's the training? What is the quality control? How are the specimens stored? What particular tests are anticipated? What is meant by temperature-controlled? 'analyzing some specimens immediately per standard antenatal care guidelines and storing others for later study-related analyses"can be unpacked.

 Results: 

 There is some redundancy in the results text when looking at the table and vice versa. Not sure how the authors can handle this. In conformity with data and specimen collection as described in methods, advised to also detail the specimens collected so far?

 Table 2: 

 Education- too many categories. Can collapse.

 GA missing on 22 women- how were they included?

 BMI-Typo, should be 13.5 not 13-5

 Discussion: Again can be realigned with the results presentation.

I confirm that I have read this submission and believe that I have an appropriate level of expertise to confirm that it is of an acceptable scientific standard, however I have significant reservations, as outlined above.

# Response

**Published:** 2018-11-12

**Authors:** Jeffrey S. A. Stringer

**Affiliations:** University of North Carolina, USA

**Fyezah Jehan**, Department of Pediatrics and Child Health, Aga Khan University, Karachi, Pakistan 

**Approved with Reservations**

Overall an important report of a biorepository cohort in LMIC, in a high prematurity setting defining baseline characteristics of the cohort at enrollment. Some recommendations for improvement :-

**Abstract:**Needs to be realigned with the article text. Methods incomplete, all salient points need to be mentioned eg. GA at eligibility, samples being stored in biorepository. Similarly, what outcome information is being collected. Conclusion mentions adverse birth outcomes although focus in the introduction has been on preterm.

*Thank you for noting these inconsistencies. We have expanded our description of assessment of birth outcomes to include not only gestational age at delivery, but also “neonatal birthweight, vital status, and sex” (Abstract, lines 17-18). We have added the gestational age criteria for enrollment (lines 14-15), as well as a description of sample collection to the methods section of the abstract (lines 18-20). Finally, we now refer to “other adverse outcomes” to the final sentence of the background of the abstract (line 7).*

**Article:**

**Introduction:**Focus of discussion has been on prematurity its risk factors and causes, while the title, methods, objectives are related to establishment of a bio repository. It isn't entirely clear if this is a description of the biro repository or a description of cohort characteristics of the women in the biorepository or both. A clearer enunciation of the objective needs to be made. Adverse outcomes to be studied are detailed in the discussion session so I would suggest to either bring that detail into the methods, in order to describe the biorepository completely.

*The purpose of this cohort description is to outline baseline characteristics of the ZAPPS Phase 1 cohort. We mention the biorepository, but that is not the primary aim of this paper. We have clarified the role of the biorepository throughout the abstract and manuscript, but note that the purpose of this paper is primarily to describe the clinical characteristics of our population.*

*The second paragraph in the discussion section (lines 268-280) details the outcomes to be studied in future analyses. We think it would be confusing to bring that detail into the methods of this paper as these analyses have not yet been conducted and we have not included any outcomes in this baseline cohort description. We have attempted to clarify this in said paragraph.*

**Methods:**

Clinical care and followup/ Data collection and management: Overall lacks specificity in discussion for e.g. what particular medical history and exam is done? How is size, neuromuscular maturity assessed? Is there a window of collection? Who does it? What's the training? What is the quality control? How are the specimens stored? What particular tests are anticipated? What is meant by temperature-controlled? 'analyzing some specimens immediately per standard antenatal care guidelines and storing others for later study-related analyses"can be unpacked.

*We have added / clarified the following:*

1. *Lines 146-149: “At each antenatal care visit, study nurses perform a vital sign assessment and a physical exam, which includes maternal height and weight; mid-upper arm circumference measurement; fundal height measurement; assessment for pallor, edema, and abdominal tenderness; fetal heart rate assessment; and cervical exam as clinically indicated.” *
2. *Lines 161-163: “Participants are asked specifically if they have ever been diagnosed with high blood pressure, heart disease, diabetes, HIV/AIDS, tuberculosis, or any other chronic illness.”*
3. *Lines 180-183: “Shortly after delivery and prior to hospital discharge, the study team documents assessment of infant vital signs, weight, length, head circumference, complete physical exam, and of neuromuscular maturity via the New Ballard Score.”*
4. *Lines 187-189: “While the 24- and 32-week study visits are scheduled according to gestational age, in the event that a participant misses her appointment, specimens may be collected as soon as possible once she returns to clinic.”*
5. *Lines 185-187: “Trained study nurses collect maternal specimens…”*
6. *Lines 186-187: “following approved standard operating procedures to ensure quality and uniformity.”*
7. *Lines 275-280: Possible protocol-related tests anticipated are described in the discussion section: ”Specimens stored at our UTH laboratory will be used for study-related analyses to identify inflammatory markers (e.g., chemokines, cytokines), microbiome community states, metabolic analytes, proteins, hormones, transcripts, and various infectious factors that may be predict adverse birth outcomes.”*
8. *Lines 191-204: “All specimens are transferred in insulated containers with continuous temperature monitoring to the on-site lab” … “All specimens are stored at -80ᵒ C in temperature-controlled freezer systems equipped with continuous temperature monitoring and text message notification of temperature deviations.”*
9. *Line 199: We have added a reference to Table 1, which delineates exactly which tests are performed immediately and we discuss which types of study-related testing may be performed in the future in the Discussion as outlined above.*

 

**Results:**

There is some redundancy in the results text when looking at the table and vice versa. Not sure how the authors can handle this. 

*We prefer to retain some redundancy between Table 1 and the Results section. Many journals require this (e.g., JAMA). The description of key baseline characteristics of our cohort in the Results section is not meant to be exhaustive, but rather to highlight key data that appear in Table 1.*

In conformity with data and specimen collection as described in methods, advised to also detail the specimens collected so far?

*While specimens have been collected, given this is a baseline paper of cohort characteristics at enrollment, we have not presented data on the total numbers of specimens that have been collected to date. This will be addressed in a future publication.*

**Table 2:**

Education- too many categories. Can collapse.

*We have collapsed this to: “None”, “0-12 years”, and “≥12 years”*

GA missing on 22 women- how were they included?

*Thank you for noting this. After additional data cleaning since our first submission of this manuscript, we have now been able to assign entry gestational age for each cohort participant.*

BMI-Typo, should be 13.5 not 13-5

*We have corrected this to “18.5” from “18-5”*

**Discussion:**Again can be realigned with the results presentation.

*Thank you. We feel that as a result of these suggested revisions, the discussion and results section are now more closely aligned.*

# Peer review: Reviewer response for version 1

**DOI:** 10.21956/gatesopenres.13888.r26410

**Published:** 2018-05-24

**Authors:** Zahida Qureshi

**Affiliations:** 1 Department of Obstetrics and Gynaecology, School of Medicine, University of Nairobi, Nairobi, Kenya

Thank you for giving me the opportunity to review the research article entitled “The Zambian Preterm Birth Prevention Study (ZAPPS: Cohort characteristics at enrollment)”

 Overall the study is important to understand the dynamics of preterm birth in a sub-saharan developing country, with intent to plan interventions.

 I have the following comments: -

 Abstract:

 Methods – should read as ‘the Zambian Preterm Birth Prevention study (ZAPPS) is an observational study, of a prospective antenatal cohort ……….’

 Results- this cohort is 1450 participants and is not clear is this the entire cohort or a subset as the protocol mentions the enrollment to be estimated at 4000, so is recruitment still ongoing? This needs to be clarified. 

 Discussion – also add the primary objective of the ZAPPS study, as stated below

 Page 3 

 Primary objective – should also include the primary objective of the study as stated in the protocol  

 Primary outcome measure – Rate of preterm births per 100 person years, incidence rate of preterm birth (delivery <37 weeks’ gestation)

 Page 4 Clinical care and follow up

 It is stated that after enrollment women return for cervical length evaluation between 20 and 24 weeks and those with a short cervix have additional visits at 28 and 32- in table 1 the ultrasound is at 34 and not 32 weeks. It is important to mention if this is standard of care for all patients at this site or only for those with previous preterm birth. Page 6 mentions that this is a non-interventional study.

 No mention is made of the maternal depression screen at 24 and 42 weeks.

 Page 4 Biological specimen collection 

 Study nurses collected maternal specimens at enrollment –and these were point of care tests for HIV, anemia, syphilis and UTI.

 The results section has a reasonably large number of missing information on some of the parameters. 

 Page 5 table 1 

 What were the vaginal and rectal swab tests for?

 Page 6 

 Results section 

 Table 2-  the missing numbers of participates with data on Syphilis is 107, Haemoglobin at enrollment 424 and malaria is 302, this needs to be explained by the authors. Patients with malaria, low haemoglobin and syphilis are at risk for preterm birth, so missing data will influence results for the ZAPPS study. 

 This should be discussed by the authors as a study limitation if these figures are correct.

 This comment does not influence this paper as it for those recruited up to Sept 2017.

 Of note is that the protocol for ZAPPS has been amended in Feb 2018 to exclude women with HIV. So eventually will there be separate analysis for the women who are included in this manuscript vs those recruited later?

I confirm that I have read this submission and believe that I have an appropriate level of expertise to confirm that it is of an acceptable scientific standard.

# Response

**Published:** 2018-11-12

**Authors:** Jeffrey S. A. Stringer

**Affiliations:** University of North Carolina, USA

**Zahida Qureshi**, Department of Obstetrics and Gynaecology, School of Medicine, University of Nairobi, Nairobi, Kenya 

**Approved**

Thank you for giving me the opportunity to review the research article entitled “The Zambian Preterm Birth Prevention Study (ZAPPS: Cohort characteristics at enrollment)”

Overall the study is important to understand the dynamics of preterm birth in a sub-saharan developing country, with intent to plan interventions.

I have the following comments: -

**Abstract:**

Methods – should read as ‘the Zambian Preterm Birth Prevention study (ZAPPS) is an observational study, of a prospective antenatal cohort ……….’

*This suggested edit has been made (line 10).*

**Results-**this cohort is 1450 participants and is not clear is this the entire cohort or a subset as the protocol mentions the enrollment to be estimated at 4000, so is recruitment still ongoing? This needs to be clarified. 

*Recruitment is indeed ongoing, but under a slightly revised protocol. This is a description of the cohort established under the original protocol only. The original estimated enrollment of 4000 has been amended to 2000 on the ClinicalTrials.gov website.*

**Discussion**– also add the primary objective of the ZAPPS study, as stated below

*Our primary objective in the protocol reads as follows: “to establish a cohort of 2,000 pregnant women and their infants, following them through delivery and up to 42 days postpartum.” We have revised the language in the Discussion of the abstract to reflect that original wording (lines 31-32).*

**Page 3**

Primary objective – should also include the primary objective of the study as stated in the protocol  

*Our primary objective in the protocol reads as follows: “to establish a cohort of 2,000 pregnant women and their infants, following them through delivery and up to 42 days postpartum. We will recruit and enroll 2,000 women and their newborn infants in Zambia.” We have revised the language in our “Primary objective” section to reflect this wording (lines 106-110).*

Primary outcome measure – Rate of preterm births per 100 person years, incidence rate of preterm birth (delivery <37 weeks’ gestation)

*Thank you for noting this inconsistency. The primary outcome as stated in the most recent protocol is as follows:*

*“The primary outcome is preterm birth, defined as birth prior to 37 weeks completed gestation. The secondary outcomes are (1) delivery prior to 34 weeks completed gestation and (2) low birth weight, defined as less than 2500 grams.”*

*We have added an “Outcomes” section to the Methods of this paper to address this omission (lines 111-118).*

**Page 4 Clinical care and follow up**

It is stated that after enrollment women return for cervical length evaluation between 20 and 24 weeks and those with a short cervix have additional visits at 28 and 32- in table 1 the ultrasound is at 34 and not 32 weeks. It is important to mention if this is standard of care for all patients at this site or only for those with previous preterm birth. Page 6 mentions that this is a non-interventional study.

*We have revised the text to the following: “After enrollment, all participants return for universal cervical length evaluation between 20 and 24 weeks’ gestation. Those with a short cervix by transvaginal ultrasound, defined as <2.5 cm, then attend additional visits scheduled at 28 and 32 weeks’ gestation for repeat cervical length ultrasounds and are referred to a study physician at the UTH for further counseling and follow-up” (lines 135-139). The additional visits at 28 and 34 weeks’ for cervical length ultrasound are only for those women with short cervix. This is explained also in the footnote to Table 1. Biometry ultrasound at 32 weeks is performed for all participants.*

No mention is made of the maternal depression screen at 24 and 42 weeks.

*Thank you for pointing this out. The following sentence has been added to the section on Clinical care & follow-up:*

*“The Edinburgh Postnatal Depression Screen is self-administered by participants at 24 weeks and again at the postpartum visit. Participants who screen positive are referred to outpatient psychiatric care” (lines 157-159). In addition, “Maternal depression screen” appears in Table 1 at 24 weeks and 42 days postpartum.*

**Page 4 Biological specimen collection**

Study nurses collected maternal specimens at enrollment –and these were point of care tests for HIV, anemia, syphilis and UTI.

The results section has a reasonably large number of missing information on some of the parameters. 

*Since our study is integrated with routine care at the clinical sites, for several cases—particularly in the early conduct of the study—we did not repeat routine tests previously performed at government clinics and failed to document these results on our study forms. This is a limitation of the study that we have described in our Discussion (lines 295-299). We also note that it has been rectified in Phase 2 of the cohort (line 299).*

                   

**Page 5 table 1**

What were the vaginal and rectal swab tests for?

*Vaginal and rectal swabs are collected for specimen storage for future protocol-related testing. No real-time tests are currently being performed on these swabs. We have clarified this in Table 1.*

**Page 6**

**Results section**

Table 2-  the missing numbers of participates with data on Syphilis is 107, Haemoglobin at enrollment 424 and malaria is 302, this needs to be explained by the authors. Patients with malaria, low haemoglobin and syphilis are at risk for preterm birth, so missing data will influence results for the ZAPPS study. 

This should be discussed by the authors as a study limitation if these figures are correct.

*These figures are correct. As we note above, since our study is integrated with routine care at the clinical sites, for several cases—particularly in the early conduct of the study—we did not repeat routine tests previously performed at government clinics and failed to document these results on our study forms. This is a limitation of the study that we have described in our Discussion (lines 295-299). We also note that it has been rectified in Phase 2 of the cohort (line 299).*

This comment does not influence this paper as it for those recruited up to Sept 2017. Of note is that the protocol for ZAPPS has been amended in Feb 2018 to exclude women with HIV. So eventually will there be separate analysis for the women who are included in this manuscript vs those recruited later?

*That is correct. Given the amended protocol has revised key inclusion / exclusion criteria, the next phase of the ZAPPS cohort has not been described in this baseline manuscript. We have added an explicit description of the two phases of the ZAPPS cohort in the Methods section (lines 102-104).*
